# The prevention of falls in patients with Parkinson’s disease with in-home monitoring using a wearable system: a pilot study protocol

**DOI:** 10.1007/s40520-022-02238-1

**Published:** 2022-09-02

**Authors:** Daiana Campani, Enrico De Luca, Erika Bassi, Erica Busca, Chiara Airoldi, Michela Barisone, Massimo Canonico, Elena Contaldi, Daniela Capello, Fabiola De Marchi, Luca Magistrelli, Letizia Mazzini, Massimiliano Panella, Lorenza Scotti, Marco Invernizzi, Alberto Dal Molin

**Affiliations:** 1 Department of Translational Medicine, University of Piemonte Orientale, Novara, Italy; 2 Health Professions’ Direction, Maggiore Della Carità Hospital, Novara, Italy; 3 Computer Science Institute, Department of Sciences and Technological Innovation, University of Piemonte Orientale, Alessandria, Italy; 4 Movement Disorders Centre, Maggiore Della Carità Hospital, Novara, Italy; 5 ALS Center, Neurology Unit, Maggiore Della Carità Hospital, Novara, Italy; 6 Department of Health Sciences, University of Piemonte Orientale, Novara, Italy

**Keywords:** Fall prevention, Wearable device, Homecare, Parkinson’s disease, Telemedicine, e-health technology

## Abstract

**Background:**

Parkinson's disease (PD) is a chronic, progressive neurodegenerative condition that gradually worsens motor function and leads to postural instability and, eventually, falls. Several factors may influence the frequency of future falls, such as slowness, freezing of gait, loss of balance, and mobility problems, cognitive impairments, and the number of previous falls. The TED bracelet is an advanced technological wearable device able to predict falls.

**Aims:**

This principal aim is to investigate the feasibility of a full-scale research project that uses the TED bracelet to identify whether individuals with PD are at risk of falling.

**Methods:**

This study will involve a pilot prospective observational study design; the subjects will include 26 patients suffering from mild PD and 26 others with no PD and no gait problems. Data will be collected from the TED bracelet and then compared to a paper-based fall diary. The enrolled participants will have a scheduled outpatient evaluation to collect both clinical and instrumental data as well as biological samples.

**Discussion:**

This pilot study could then be implemented in a larger form to further evaluate the effectiveness of the TED device. Finally, it will help further develop gait monitoring systems for people with Parkinson's disease and other neurodegenerative diseases that can affect physical function and mobility, such as dementia and Alzheimer's.

**Conclusions:**

Preventing falls and their complications could lead to major advancements in the quality of home care for patients with PD, which would significantly impact the quality of life of both these patients and their caregivers.

## Introduction

Parkinson's disease (PD) is a chronic, and progressive neurodegenerative condition that is caused by the loss of neurons that produce dopamine, the chemical responsible for communication between motor neurons [1, 2]. Though the exact causes of neurodegeneration are not yet fully understood, several factors do need to be considered. In addition to the classical motor phenotype, which is characterized by bradykinesia, rest tremors and postural instability, patients often complain of several non-motor-related symptoms, including cognitive impairment, depression, hallucinations, gastrointestinal dysfunction, and sleep disturbances, all of which have a significant impact on both patients’ and their caregivers’ lives [3].

PD is the second most common neurodegenerative disease, after Alzheimer's-caused dementia; the prevalence of PD varies globally; estimates range from 15/100,000 inhabitants of China to 150–200/100,000 inhabitants of Europe and North America. In Italy, PD has a prevalence rate of 193.7/100,000; about 5% of these individuals are under the age of 50, while 70% are over the age of 65 [4, 5].

Onset occurs at an average of around 60 years of age, and the disease is slightly more frequent in males, with a 1.5–2 higher rate of incidence. In terms of prevalence, the disease occurs in 1–2% of those over 60 and 3–5% of those over 85. Due to the increasing age of the general population, the prevalence of the disease is estimated to double by 2030 [5, 6]. Dopaminergic treatment has shown clinical benefits at early stages of Parkinson’s, but several complications can occur as the disease progresses, including motor fluctuations, postural deformities, and cognitive decline, which respond less to current therapeutic options. Also, the effect of rehabilitation on reducing dyskinesia was studied [7].

Some complications, such as postural instability and dementia, have a mean onset time of approximately 5 and 10 years from diagnosis, respectively, although this is highly variable due to individual characteristics [8]. Previous studies have identified several risk factors for future falls in patients with Parkinson’s, such as slowness, freezing of gait, loss of balance and mobility problems, cognitive impairments, and a history of previous falls [9]. Falls occur frequently in patients with Parkinson’s; incidence rates vary from 35 to 90% for falls and from 18 to 65% for recurrent falls [10]. PD patients are also prone to near-falls; main risk factors of these include jerky movements and postural instability [11]. It has been estimated that 30% of people over 65 years old experience one fall per year [12], and it is also worth noting that day-to-day activities can represent risk factors for falls because they can be influenced by reduced motor ability [13].

The results of an umbrella review suggest that in PD patients’ virtual reality rehabilitation improves gait performance [14], thus a possible reduction in the risk of falling as well. In other patient population, the use of the telemedicine and e-health technology has been described [15–20].

**Fig. 1:**
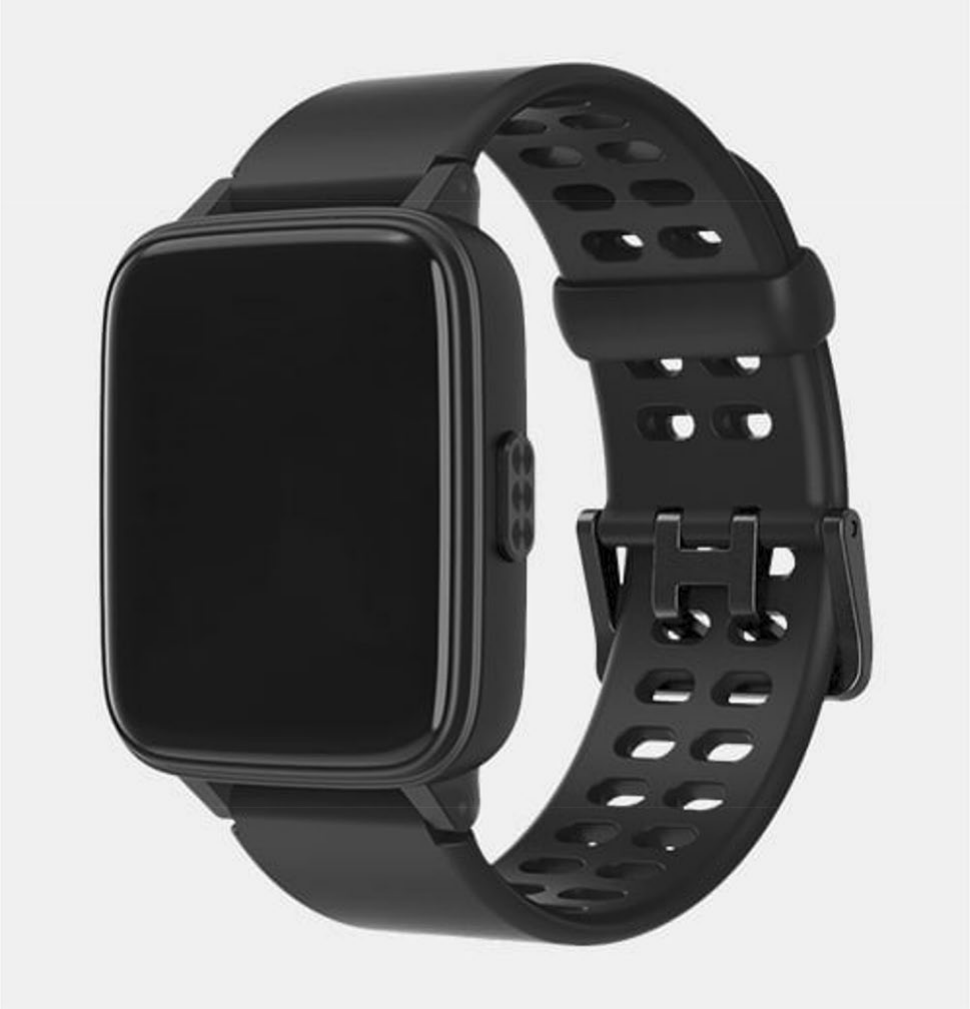
The bracelet

The use of sensors that can quickly identify the event of a fall has been documented in the literature [21]. The bracelet (see Fig. 1) in question here (called TED by the research team to facilitate communication with patients) is an advanced technological device developed by the 4Sec company (www.4secsrl.com) that is equipped with an accelerometer capable of detecting up to 200 samples per second concerning the acceleration of gravity across the x, y, and z axes. The device was developed to adapt to an individual’s biological variables. As such, it is totally programmable and its performance can be optimized for each patient. All data received by the device are sent to the cloud collection system, both continuously (with the goal of monitoring the function of interest) and in cases of specific events included in the monitoring plan, such as a fall. The data are then processed by machine learning systems that can extrapolate elements of knowledge and learning of the device from the data, using the most modern deep learning algorithm. The device shown to be sensitive to breathing movement in infants [22]

Although fall detection and prevention has been a research topic since the mid-2000s, there is a gap in the literature regarding studies of PD fall prevention systems, specifically when applied to individuals in their homes.

This study aims to investigate the feasibility of a full-scale research project that uses the TED bracelet to identify whether individuals with Parkinson’s disease are at risk of falling. Specifically, the study will have the following objectives:

- to estimate the prevalence of falling among PD patients;
- to evaluate study participants’ adherence to the device, gait characteristics and their variations, the adequateness of diary keeping;
- to compare a group of patients with PD’s gaits and risk of falling with those of a group of older citizens who are not affected by any gait or mobility altering conditions;
- to collect and store biomaterial and clinical information and store it in the Biobank of Università of Piemonte Orientale (UPO Biobank) for further research.

## Methods

### Study design

This will be a pilot prospective observational study.

### Study setting and participants

The study will be conducted at both at the Movement Disorders’ Center (Neurological Unit) of the University Hospital “Maggiore della Carità” (Novara, Italy) and at the homes of the enrolled patients.

### Inclusion and exclusion criteria

The inclusion criteria for PD patients will be: a) subjects > 65 years old suffering from Parkinson’s at an intermediate stage as defined by Hoeen and Yahr (H&Y) 1.5–3; b) the ability to understand and provide informed consent; c) preserved cognitive skills, tested as defined by a mini mental state examination (MMSE) score of > 24.

The inclusion criteria for healthy controls without gait problems will be: a) subjects > 65 years old; b) not suffering from any disease that could alter their gait; c) ability to understand and provide informed consent; c) preserved cognitive skills, as defined by a MMSE score of > 24.

The following conditions will be the exclusion criteria:

a) hospitalized subjects or nursing home residents; b) bedridden subjects; c) PD patients undergoing advanced therapy (apomorphine infusions, deep brain stimulation implants, levodopa carbidopa intestinal gel treatments); d) subjects with severe choreic dyskinesias; e) subjects with a concomitant disease that could increase their risks of falling (e.g., neuropathies, osteopenia, osteoporosis); f) subjects with neurological disorders other than idiopathic PD; g) subjects affected by atypical Parkinsonism, psychiatric disorders, or any other condition that, in the researchers’ opinion, could compromise the eligibility for the present study (e.g., having an implanted pacemaker).

### Participants’ recruitment

Participants will be recruited at the Movement Disorders Center (neurological unit) by the principal investigator and neurologists from the team. Any subject that meets the inclusion criteria will be included in the study cohort and followed up with for a maximum of 12 months. The enrollment phase will last about three months.

The investigators will provide any information required by the eligible participants to define the purposes, methods, device features, risks, and benefits of the study, and will also offer useful contacts. Participants will be invited to sign the informed consent form and to give an additional consent for the collection of peripheral blood for biobanking at the UPO Biobank. This will not affect their potential involvement in the study.

### Sample size calculation

After setting a first type error of 0.10 (pilot study), a prevalence of falls of 0.10, and a half-width of the confidence interval of 0.10, we were able to define the sample size of this study as 26 PD subjects. Each PD subject will be matched with an older citizen who is unaffected by any gait-altering conditions.

## Data collection

### Enrollment phase

At the enrollment, the following information will be collected/assessed using a datasheet:

- Age, gender, work, education level, size of family, and daily life habits (for example, whether he/she lives alone);
- Characteristics of the home;
- Pharmacological treatments;
- Comorbidities;
- Cognitive function (MMSE) [23];
- Motor function using the Movement Disorder Society’s revision of the United Parkinson’s Disease Rating Scale (MDS-UPDRS) and related subscales [24];
- Quality of life (Quality of life in Parkinson’s disease patients—PDQ-39-IT) [25];
- Fear of falling (Falls Efficacy Scale International—FES-I) [26];
- Motor and movement disorders (freezing of gait questionnaire) [27].

### Monitoring phase

The monitoring phase will last a maximum of 12 months; all information will be collected both electronically (through the TED device (Table 1)) and manually by falls diary. Study participants will be instructed to complete a diary recording on days where falls or near-falls occur. The following information will also be collected: a) time and place of the fall or near-fall; b) caregiver (or family member) witnesses; c) causes as well as physical, personal, and environmental conditions; d) activities undertaken; e) description of the dynamics of the falls/near-falls; f) consequences.

**Table 1:** Gait characteristics monitored by TED

Gait characteristics	Variables	Unit of measurement	Notes
Step cadence	Numeric	Average steps/minute	Estimated average cadence every 10 min
Estimated walking speed	Numeric	Meters/second	Estimated average speed every 10 min
Swing	Episode	TBD	Evaluation of swinging amplitude Database timestamp event record
Gait festination	Episode	Timestamp	Database timestamp event record
Fall^*^/Near-Fall^**^	Episode	Timestamp	Database timestamp event record

^*^Definition of a fall: "To inadvertently reach the ground, floor, or other lower level, excluding intentional changes in position to lean against furniture, walls, or other objects" [28]

^**^Definition of a near-fall: "Stumbling, sliding, or missteps involving a loss of balance that does not translate into a fall because corrective action is taken to recover balance" [29, 30]

The TED device will automatically record the characteristics of the participants’ gaits during the study period. At the end of the monitoring phase, the dataset will be accessible to authorized researchers. Table 2 shows the study time schedule.

**Table 2:** Study Time Schedule

	Study period			
	Movement disorder center		Home	Close-out
Timepoint	*-t*_*1*_	*t*_*0*_	*t*_*1*_* (12 months)*	*t*_*2*_
**Enrollment**				
Eligibility screen	**X**			
Informed consent	**X**			
Device delivery and instruction provision		**X**		
**Monitoring phase**				
TED device				
Falls diary				
**Assessment**				
*Clinical & lifestyle*				
Demographics	**X**			
Daily lifestyle habits	**X**			
Home features	**X**			
Pharmacological treatment	**X**			
Comorbidities	**X**			
MMSE	**X**			
MDS-UPDRS	**X**			
PD-39-IT	**X**			
FES-I	**X**			
Freezing of Gait Qtn	**X**			
*Gait characteristics*				
Step cadence				
Estimated walking speed				
Swing				
Gait festination				
Fall/near-fall				
Falls^$^ Characteristics				
Gait pattern				**X**

^$^Only in case participants will fall, additional information will be collected (time and place, caregiver witnessing, consequences, etc.)

### TED device

The TED device is safe, assembled in a shock-resistant plastic case, and is very small and easily transportable. The radio transmission complies with the regulations in force in terms of EMC emissions. Although the device's sensors possess a high level of adequacy and sensitivity (except when it comes to the measuring of falls), the application to detect the near-falls, the measurement of the cadence of the step, of the walking speed, and the oscillation was used only in the context of experimental testing.

### Study outcomes

The following outcomes will be considered:

- The number of falls and near-falls that occurred during the study will be counted using numbers recorded by both the TED bracelet and the participants’ diaries. The double counting will verify data coherence between TED device recordings and the fall events reported in the diary. Furthermore, the adequacy of diary keeping will be verified too.
- Consistent use of the TED devices will be measured as follows. The absence of gait characteristic measurements for a period exceeding 24 h will be considered an interruption in the use of the device. The interruption will be considered temporary if the use of the device is resumed by the end of the observation period and final if it is not. In the event of a temporary interruption, the number of outage events that occurred during the observation period will be counted.
- The gait characteristics associated with falls and near-falls in Parkinson’s patients will also be examined. At the end of the observation period, the TED device's actual data transmission will be evaluated, and the trends in the parameters (pace, walking speed, oscillation, festination) will be analyzed. Subsequently, these data will be related to the falls / near-falls.

## Statistical analysis

Descriptive statistics will be utilized to describe patients’ characteristics. Mean and standard deviation (SD) will be used to describe continuous variables with normal distribution. Continuous variables that are not normally distributed will be expressed using median and interquartile range. Absolute and relative frequencies will be presented for categorical variables.

Consistent use of the device will be defined as the ratio of the number of subjects who never experienced interruption to the total number of participating subjects and by counting the number of interruptions each individual experiences. For the first indicator, the confidence interval will be 95%. Diary-keeping adequacy will be assessed by calculating the proportion, and the corresponding 95% CI, of the number of days where the diary was correctly filled in as compared to the number of days on which the bracelet was worn. Cohen’s Kappa will be used to assess the level of agreement between the TED device and the diary in terms of fall detection. Units of interest will be days (or daily time bands); data points will include either both instruments identifying a fall (agree) or one recording an event that the other has not (disagree). The clinician and the subject will resolve any unclear cases by comparing the data from the TED device with the diary. Trends in gait patterns will be displayed graphically and analyzed through specific models for repeated measurements. The gaits' characteristics (exposure) will be evaluated for various time frames of different lengths to represent the period before the fall/near-fall (outcome).

Logistic regression models will be also used to verify the association between the variables used to determine gait patterns and the risk of falling. Furthermore, the predictive power of each variable on the risk of falling will be determined by calculating the mean values of various parameters during the 15, 30, 45, and 60 min leading up to the fall, for the events, and in the same time frame, for the non-events, identified randomly during the observation period.

Specific conditional logistic regression models will be used to compare the association between gait patterns and risk of falling between individuals suffering from Parkinson's disease and individuals not suffering from any pathologies that could alter gait. In these models, in addition to the variables used to determine the patterns, a variable identifying the disease state (Parkinson’s vs. absence of a pathology that could alter gait) and an interaction term between pattern and disease state will be included.

## Discussion

Twenty-six patients with intermediate PD who are being treated at the Movement Disorders’ Center, and 26 participants over 65 years old not affected by diseases that could alter their gait (the control group) will be enrolled in the pilot study. The results of this study could be helpful in the evaluation of the feasibility of a more extensive study.

This study will be the first to be conducted using the TED device in PD patients. However, other studies using similar devices have shown promising results [21, 31], leading us to believe in the potential of the TED device. Notably, the TED device is a bracelet that can be worn with a level of comfort that is similar to a bracelet or watch. Therefore, we expect a high level of wearability (adherence), acceptability, and gradeability from the subjects. This device is also easy to use and can be operated independently by either the user or caregiver, meaning that people living alone can be monitored while maintaining their independence and privacy. This study could be useful in implementing a gait monitoring system in practice for people with PD or other neurodegenerative diseases that affect physical function and mobility, such as Alzheimer’s dementia. Preventing falls and their related issues (e.g., disability) could have a significant impact on both PD patients’ and their caregivers’ quality of life.

Last, this study highlights biobanking’s key role in fostering scientific research by allowing for the discovery and validation of disease markers and novel therapeutic strategies, the adherence to standard laboratory practices, and the quality of results and ethical requirements [32]. Furthermore, biobanking encourages both government agencies and international infrastructure to recognize the best ethical, scientific, and legal practices and guidelines for providing healthcare [33]. Thus, it effectively supports health studies, not solely via relevant biospecimens collection linked to relevant personal and health information (health records, family history, lifestyle, genetic information), but via the implementation of new factors like imaging biobanks and data on diagnostic interventions [32, 33].

This study does not include the elderly population who use walking aids and wheelchairs.

The TED device can detect movement in space—if it is worn on the wrist, it will detect one's position based on where one's wrist is. The device is able to detect a wheelchair user's fall, but for accurate detection, it would have to be worn at chest level. In this case, the recorded fall time would be shorter than for a person who fell while standing. It is necessary to establish a person's basic posture beforehand. In the case of a wheelchair, the parameters of the algorithm that detect the fall would have to be changed according to the sitting position. From our experience, we believe that the most suitable device for detecting falls in wheelchair users is one worn on the chest. We are not aware of any published studies on wheelchair users. The detection of falls in wheelchair users and people using walking aids could be a topic for further research.

Should the results of our project support the validity of the TED device in its ability to identify gait patterns predictive of falling or near falling, the device could be used to stratify the risk level in frail patients at high risk of fracture (e.g., osteoporotic). The device could also be used in the general population to establish risk factors for falls and near-falls, both in the home and in structured care settings (e.g., residential facilities and hospitals).

To complement the assessment carried out by the TED device, it is considered appropriate to combine an assessment of home risk factors [34]. Further fall prevention interventions could be assessed and implemented based on the physical capabilities of the assessed person [35]. For example, exercise is one such intervention [36].

This study’s protocol may have a limitation in its design that would make it impossible to determine a priori if the overall cost of the monitoring system is convenient and sustainable over time.

In conclusion, the development of a gait monitoring system for people with PD or other neurodegenerative diseases that impact physical functions and mobility, such as dementia or Alzheimer’s is undoubtedly useful. Preventing falls will offer a major advancement in home care assistance for both patients with Parkinson’s disease and their caregivers, significantly improving their quality of life.

## Declarations

### Confidentiality

The promoters of the study and UPO Biobank are engaged in protecting the sensitive, clinical, and non-clinical personal data of the subjects involved in the study as established by international and national legislation and in the UPO Biobank code of ethics.

### Dissemination policy

The study promoter will be responsible for publishing the data resulting from the study. The dissemination of data through scientific publications and / or presentation in congresses, conferences and seminars will take place in anonymous and aggregate form.
